# The oncogenic role of LncRNA FAM83C-AS1 in colorectal cancer development by epigenetically inhibits SEMA3F via stabilizing EZH2

**DOI:** 10.18632/aging.103835

**Published:** 2020-10-27

**Authors:** Weinan Xue, Fan Wang, Peng Han, Yanlong Liu, Bomiao Zhang, Xinyue Gu, Yue Wang, Mingqi Li, Yashuang Zhao, Binbin Cui

**Affiliations:** 1 Department of Colorectal Surgery, Harbin Medical University Cancer Hospital, Harbin 150081, China; 2 Department of Epidemiology, School of Public Health, Harbin Medical University, Harbin 150081, China; 3 Department of Pharmacology and Toxicology, Wright State University, Fairborn, OH 45435, USA

**Keywords:** SEMA3F, colorectal cancer, FAM83C-AS1, EZH2, ZRANB1

## Abstract

Inactivation of Semaphorin 3F (SEMA3F) is involved in colorectal cancer development. However, the mechanism by which SEMA3F is regulated remains elusive. Deregulation of lncRNAs have been implicated in multiple human malignancies, including colorectal cancer (CRC). To date, it is still unclear whether and how lncRNA regulates SEMA3F expression and mediates CRC progression. Here we identify the oncogenic role of lncRNA FAM83C antisense RNA 1 (FAM83C-AS1) in CRC. FAM83C-AS1 is upregulated in tumor tissues and cells of CRC, which is negatively correlated with SEMA3F expression. Reciprocally, knockdown of FAM83C-AS1 exhibits inhibitory effects on the malignant transformation of CRC. Moreover, our data uncover that FAM83C-AS1 enhances methylation of SEMA3F promoter H3K27me3 via upregulating methyltransferase enhancer of zeste 2 polycomb repressive complex 2 subunit (EZH2). Specifically, FAM83C-AS1 stabilizes EZH2 protein through recruiting the zinc finger RANBP2-type containing 1 (ZRANB1). Both *in vitro* and *in vivo* rescue assays exhibit that SEMA3F is dispensable for the tumor-promoting effects of FAM83C-AS1 on CRC progression. Our data thus demonstrate that the epigenetic role of FAM83C-AS1 in suppression of SEMA3F expression through stabilization of EZH2 to drive CRC progression, which may be conducive to discovering novel therapeutic targets for the treatment of CRC.

## INTRODUCTION

As one of the most prevalent malignant tumor around the world, the mortality rate of colorectal cancer (CRC) is increasing, which caused approximately 694,000 deaths annually [1]. The progression of CRC is a multifactorial disease process, involving germline and somatic mutations, especially some pivotal oncogenes or tumor suppressor genes [2, 3]. Recent progress in early detection and intervention strategies has improved the overall survival rate of patients with CRC to some extent. Unfortunately, due to high rate of recurrence and metastasis after surgery, unfavorable prognosis poses a severe threat to patients with advanced CRC [4–6]. Tumor metastasis, the primary factor leading to therapeutic failure among patients with advanced CRC, is involved in the mechanism of tumor cells acquiring enhanced invasive capacity and spreading to remote organs [7, 8]. Over the past years, numerous efforts have been made to understand the pathogenesis of CRC so as to develop more effective therapies for patients with CRC. As a result, novel therapeutic methods, such as immunotherapy and targeted molecular therapy, have been adopted in clinical practice, which has significantly improved the prognosis [9]. Nonetheless, molecular mechanisms involved in the development and progression of CRC are particularly complicated and aberrantly expressed genes have been closely related to biological processes leading to CRC progression [10, 11]. Therefore, further exploration of the underlying mechanism will be of great value to change the landscape of treatment for CRC.

Genomic and transcriptomic sequencing has revealed that human genome may be transcribed into two types of RNA molecules: one is noncoding RNAs (ncRNAs) and the other is protein-coding mRNAs [12]. Unlike mRNAs that serve as template for protein synthesis, ncRNAs participate in the biological processes and function directly in the form of RNA [13]. In addition, ncRNAs can be further classified into different subclasses according to their length and function. Long ncRNAs (lncRNAs) are identified as a group of ncRNAs with a length of over 200 nucleotides [14]. Additionally, lncRNAs are functionally defined as transcripts that possess little or no protein-coding potential, which is largely due to lack of an open reading frame [12, 15]. Existing evidence has demonstrated that lncRNAs are involved in cancer progression through interacting with a variety of signaling molecules [14, 16]. Studies have revealed that lncRNAs act as a crucial regulator of tumorigenesis by eliciting biological effects along with regulatory mechanisms in various human cancers, owing to their interactions with a variety of other molecules. LncRNAs play a key role in numerous cellular events, including chromatin modification, gene regulation, transcriptional and post-translational regulations [17]. Competing endogenous RNA (ceRNA) and RNA binding proteins (RBPs) are the key factors involved in most prevalent post-transcriptionally regulatory mechanisms of lncRNAs in human cancers, including CRC. For example, lncRNA HIF1A-AS2 mediates CRC progression through miR-129-5p/DNMT3A (DNA cytosine-5-methyltransferase 3A) axis [18]; LncRNA PCA3 promotes prostate cancer progression through sponging miR-218-5p and modulating HMGB1 (high mobility group box protein 1) [19] and LncRNA OCC-1 suppresses cell growth by destabilizing HuR protein in CRC [20]. Moreover, other common mechanisms were also involved in tumor progression. For instance, lncRNA SLCO4A1-AS1 promotes tumor cell proliferation and metastasis via activating the β-catenin-dependent Wnt pathway in CRC [21]. LncRNA HOXB13-AS1 promotes glioma progression by regulating HOXB13 gene methylation through EZH2 [22].

Based on the results of previous studies, semaphorin 3F (SEMA3F) has been proved to have inhibitory effect on cell proliferation, metastasis as well as stemness in CRC [23, 24]. Given that lncRNAs can mediate the progression of human malignancies by regulating mRNA expression transcriptionally or post-transcriptionally, we attempted to explore the potential lncRNA that mediates disease progression by regulating SEMA3F expression in CRC. This study was aimed to identify the lncRNA that interacts with SEMA3F in CRC and unveil the underlying molecular mechanisms of disease progression.

## RESULTS

### Opposite effects of FAM83C-AS1 and SEMA3F in colorectal cancer development

**Figure 1:**
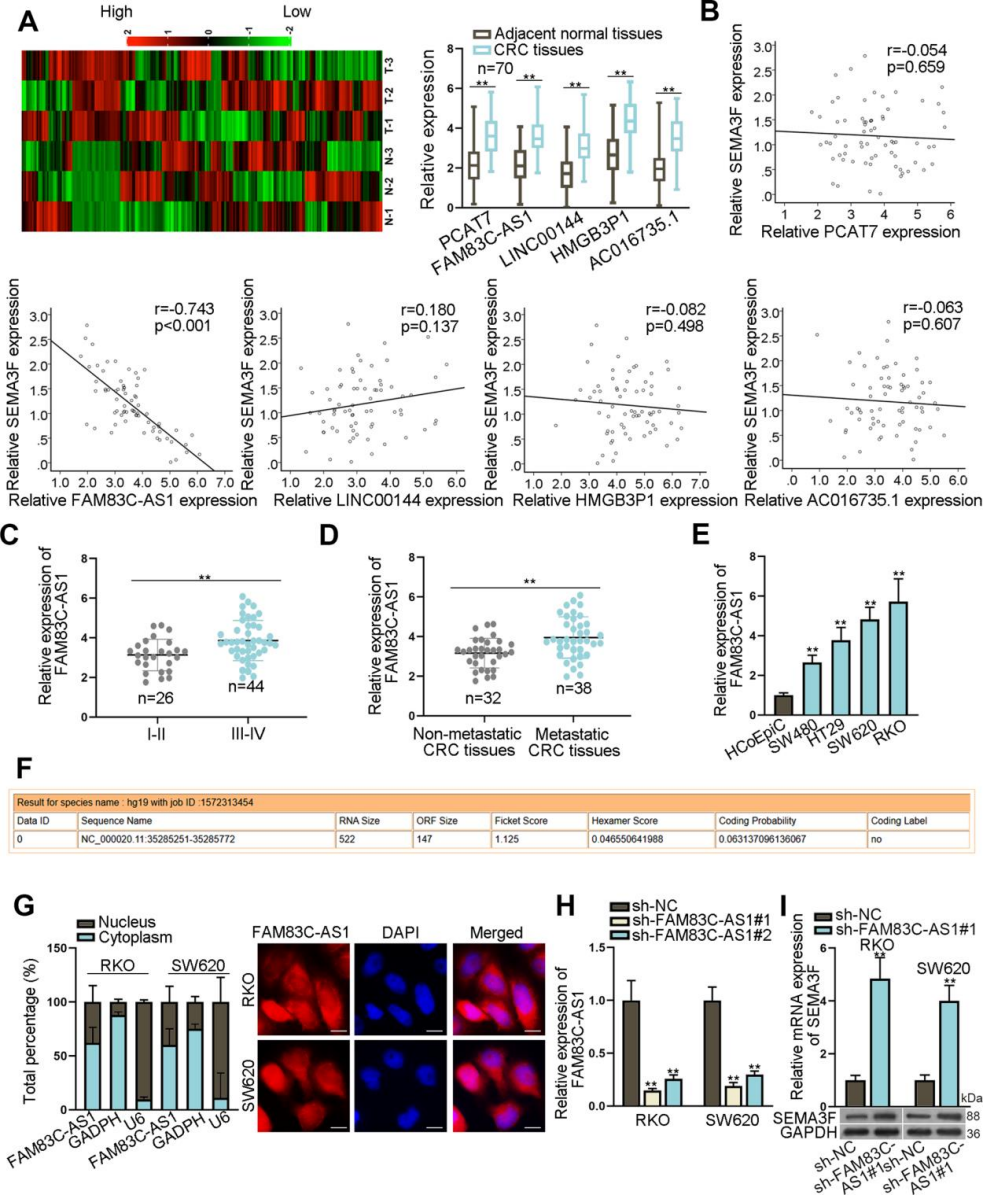
**Opposite effects of FAM83C-AS1 and SEMA3F in CRCs**. (**A**) The expression of 242 lncRNAs in CRC tissues and corresponding non-tumor tissues was detected using microarray analysis, and the expression of PCAT7, FAM83C-AS1, LINC00144, HMGB3P1 and AC016735.1 was detected in CRC tissues and adjacent non-tumor tissues using RT-qPCR. (**B**) The correlation between SEMA3F and lncRNAs (PCAT7, FAM83C-AS1, LINC00144, HMGB3P1 and AC016735.1) was analyzed using Spearman’s correlation analysis. (**C**) FAM83C-AS1 expression was detected using RT-qPCR in early and advanced stages of CRC patients. (**D**) FAM83C-AS1 expression in metastatic CRC tissues and non-metastatic CRC tissues was detected using RT-qPCR. (**E**) FAM83C-AS1 expression in CRC cell lines and normal human colonic epithelial cell line (HCoEpiC) was examined by RT-qPCR. (**F**) The coding potential of FAM83C-AS1 was obtained from CPAT. (**G**) The subcellular localization of FAM83C-AS1 was determined by subcellular fractionation and FISH assays. (**H**) The efficiency of FAM83C-AS1 was evaluated using RT-qPCR. (**I**) SEMA3F expression in RKO and SW620 cells transfected with sh-FAM83C-AS1#1 or sh-NC was detected using RT-qPCR and western blot analyses. ^**^P < 0.01, ^***^P < 0.001.

In the attempt to determine the candidate lncRNA that contributes to CRC progression, we first explored the Gene Expression Omnibus GEO Database (https://www.ncbi.nlm.nih.gov/gds/) and identified 242 lncRNAs that were upregulated in CRC tissues under specific conditions (threshold: adjusted *P* < 0.01, logFC > 2) (Supplementary Table 1). To further screen out the desired lncRNAs, we utilized RT-qPCR assay to identify the top 5 lncRNAs (PCAT7, FAM83C-AS1, LINC00144, HMGB3P1 and AC016735.1) that were remarkably upregulated in CRC tissues as compared to corresponding non-tumor tissues (Figure 1A). To identify the lncRNA that could interact with SEMA3F in CRC, we then used the Spearman’s correlation analysis of the gene expression data. As shown in Figure 1B, only the expression of FAM83C-AS1 was negatively correlated with the expression of SEMA3F. Moreover, the expression of FAM83C-AS1 in patients with advanced CRC was higher as relative to those in CRC patients at earlier stages (Figure 1C). In addition, a significantly higher expression of FAM83C-AS1 was observed in metastatic CRC tissues in contrast to non-metastatic CRC tissues (Figure 1D). Interestingly, through analyzing the mRNA level by RT-qPCR assay, we found that CRC cell lines, particularly RKO and SW620 cells, expressed high level of FAM83C-AS1 as compared with normal intestinal cell line (HCoEpiC) (Figure 1E).

To study the subcellular localization of FAM83C-AS1, we first designed the specific probes for FAM83C-AS1 through the Coding-Potential Assessment Tool (CPAT) (http://lilab.research.bcm.edu/cpat/index.php) (Figure 1F). Afterwards, subcellular fractionation and FISH assays were conducted in RKO and SW620 cells. As shown in Figure 1G, the distribution of FAM83C-AS1 was a bit denser in nucleus than in cytoplasm. We then used siRNAs to specifically silenced the expression of FAM83C-AS1 in RKO and SW620 cells (Figure 1H). Notably, the expression of SEMA3F was dramatically increased along with down-regulation of FAM83C-AS1 (Figure 1I). Taken together, the expression of FAM83C-AS1 is selectively increased in CRC tissues, which is negatively correlated with SEMA3F expression.

### Knockdown of FAM83C-AS1 suppresses the progression of CRC

**Figure 2:**
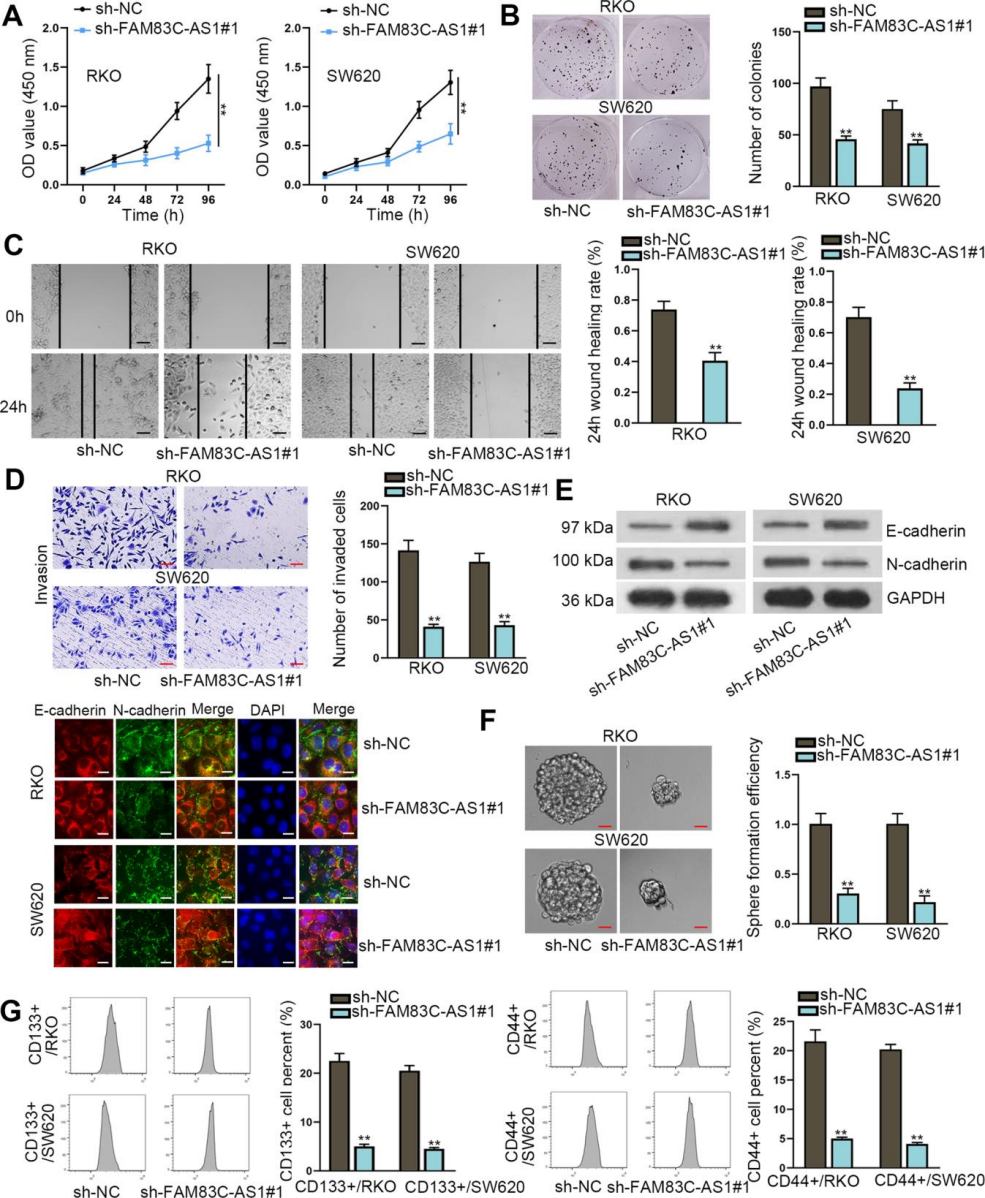
**Knockdown of FAM83C-AS1 suppresses CRC progression.** (**A**, **B**) Cell proliferation was evaluated by CCK-8 and colony formation assays. (**C**, **D**) Wound healing and transwell assays were carried out to measure cell migration and invasion abilities in different groups. (**E**) EMT process in transfected cells was analyzed using western blot and immunofluorescence analyses. E-cadherin and N-cadherin were found to be EMT-related proteins. (**F**) The sphere formation ability of transfected cells was assessed using sphere formation assay. (**G**) Cell stemness in different groups was analyzed through flow cytometer. CD133^+^ or CD44^+^ was utilized as stem cell marker and was related to cell stemness. ^**^P < 0.01.

To study the role of FAM83C-AS1 in the development of CRC, we silenced the endogenous FAM83C-AS1 in RKO and SW620 cell lines. As illustrated in Figure 2A and 2B, silence of FAM83C-AS1 limited the proliferation of RKO and SW620 cells as relative to control cells. We then used Transwell and Wound-healing assays to assess the role of FAM83C-AS1 in the invading and migrating abilities of CRCs. As shown in Figure 2C and 2D, downregulation of FAM83C-AS1 curbed the invasion and migration of RKO and SW620 cells compared with cells transfected with scramble. To determine whether FAM83C-AS1 deficiency suppresses the biological process of epithelial-mesenchymal transition (EMT), we employed western blot and immunofluorescence staining assays. As shown in Figure 2E, higher amount of E-cadherin and lower amount of N-cadherin were detected in cells in absence of FAM83C-AS1. Furthermore, we also detect whether FAM83C-AS1 affects the colonic ability of tumor. As shown in Figure 2F, compared with control cells, FAM83C-AS1 depletion exhibited significant tumor suppressive effect on the sphere formation ability of tumor cells. Consistent to this result, the percentages of CD133^+^ and CD44^+^ cells were found to decrease with the knockdown of FAM83C-AS1 in RKO and SW620 cells marked with CD133^+^ or CD44^+^, indicating that downregulation of FAM83C-AS1 blocks the cancer stemness (Figure 2G). In summary, we identify the oncogenic role of FAM83C-AS1 in CRC progression.

### FAM83C-AS1 inhibits SEMA3F expression in an EZH2-dependent manner

**Figure 3:**
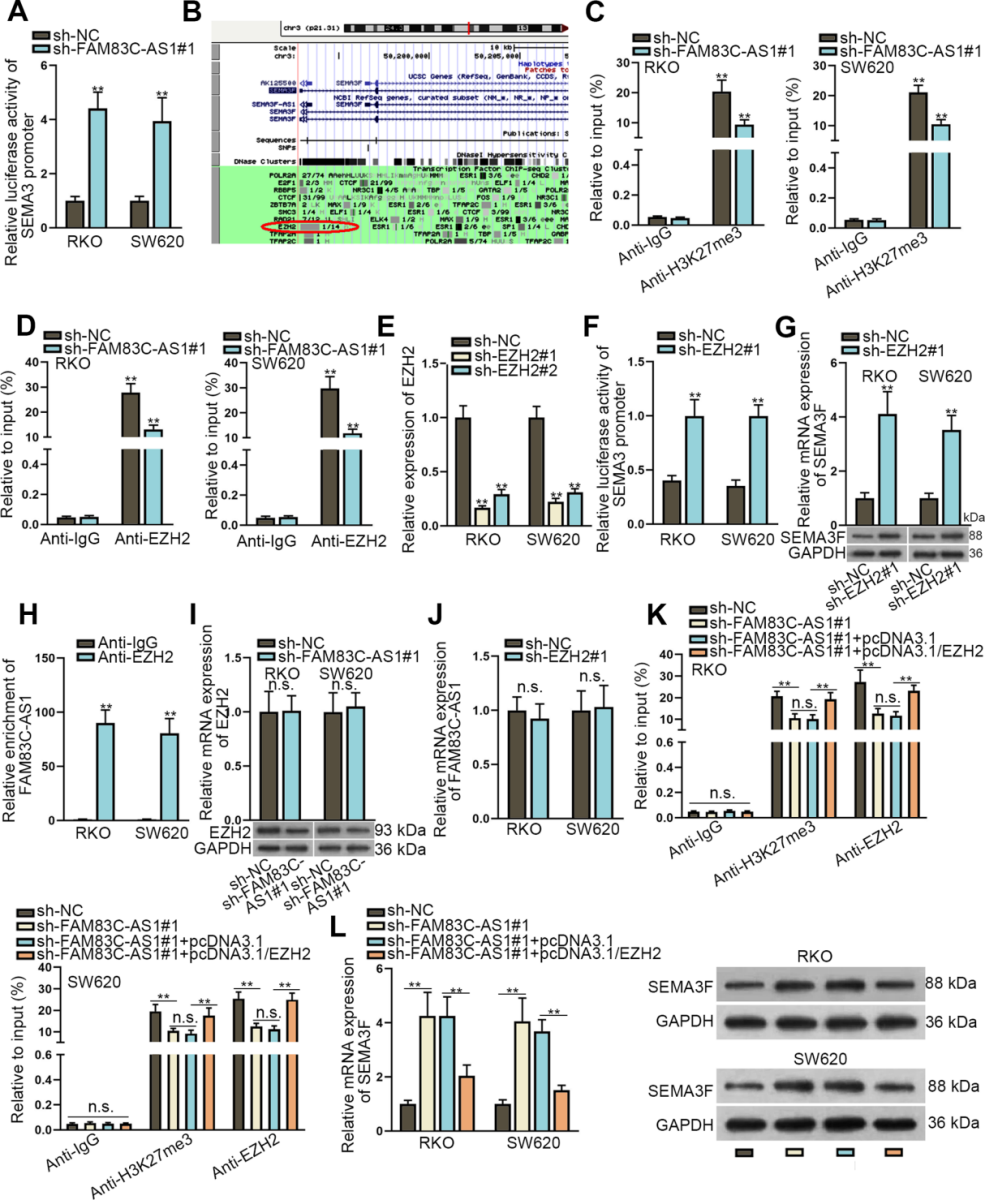
**FAM83C-AS1 inhibits SEMA3F expression through interacting with EZH2 in CRC tumor cells.** (**A**) The interaction between FAM83C-AS1 and SEMA3F promoter was tested by luciferase reporter assay. (**B**) SEMA3F was speculated to boast the potential of binding with EZH2 via UCSC. (**C**, **D**) H3K27me3 occupancy and EZH2 binding of the SEMA3F promoter in transfected cells were analyzed using ChIP. (**E**) The efficiency of EZH2 knockdown was detected using RT-qPCR. (**F**) The interaction between EZH2 and SEMA3F promoter was investigated using luciferase reporter assay. (**G**) RT-qPCR and western blotting assays were conducted to detect the mRNA and protein expression of SEMA3F after RKO and SW620 cells were transfected with sh-EZH2#1 or sh-NC. (**H**) RIP assay demonstrated that SEMA3F could bind to EZH2 in RKO and SW620 cells. (**I**) RT-qPCR and western blotting analyses were used to detect the mRNA and protein expression of EZH2 in RKO and SW620 cells transfected with sh-FAM83C-AS1#1 or sh-NC. (**J**) FAM83C-AS1 expression in RKO and SW620 cells transfected with sh-EZH2#1 or sh-NC was determined using RT-qPCR. (**K**) H3K27me3 occupancy and EZH2 binding to SEMA3F promoter in transfected cells were analyzed using ChIP. (**L**) mRNA and protein expression of SEMA3F in RKO and SW620 cells transfected with different plasmids were examined using RT-qPCR and western blotting analysis. ^**^P < 0.01. n.s.: no significance.

To further explore the underlying mechanism involved in the potential regulatory effect of FAM83C-AS1 on SEMA3F in CRC, we used the luciferase reporter assay. As shown in Figure 3A, knockdown of FAM83C-AS1 suppressed the luciferase activity of SEMA3F promoter, indicating that FAM83C-AS1 regulated the transcription level of SEMA3F. Based on the efforts of exploring the genome bioinformatics site the UCSC (http://genome.ucsc.edu/), we discovered that SEMA3F possessed the potential of binding to EZH2 (Figure 3B). Thus, it was speculated that FAM83C-AS1 might regulate trimethylation of histone H3 lysine 27 (H3K27) (H3K27me3) of SEMA3F promoter. To further confirm these results, we used ChIP assays and found that FAM83C-AS1 knockdown decreased H3K27me3 occupancy at the promoter of SEMA3F and EZH2 binding to the promoter of SEMA3F (Figure 3C and 3D). To investigate the role of EZH2 in this process, we used siRNA to silence the endogenous EZH2 in RKO and SW620 cells (Figure 3E). As shown in Figure 3F, the luciferase activity of SEMA3F promoter was significantly enhanced in EZH2-silencing cells as relative to control cells. Accordingly, knockdown of EZH2 augmented both the mRNA and protein level of SEMA3F (Figure 3G). Subsequently, the result of RIP assay demonstrated that SEMA3F bound to EZH2 in RKO and SW620 cells (Figure 3H). Later on, through analyzing by RT-qPCR and western blot assays, FAM83C-AS1 depletion had no distinct impact on mRNA expression of EZH2 but downregulated the protein expression of EZH2 (Figure 3I). Besides, EZH2 knockdown exhibited no observable effect on the expression of FAM83C-AS1 (Figure 3J). Further, the decreases in H3K27me3 occupancy at SEMA3F promoter and EZH2 binding to SEMA3F promoter induced FAM83C-AS1 knockdown were reversed by EZH2 upregulation (Figure 3K). Moreover, EZH2 overexpression facilitated FAM83C-AS1-mediated downregulation of the mRNA and protein level of SEMA3F (Figure 3L). Taken together, FAM83C-AS1 suppresses SEMA3F expression through interacting with EZH2 in CRCs.

### FAM83C-AS1 stabilized EZH2 protein via binding to ZRANB1

**Figure 4:**
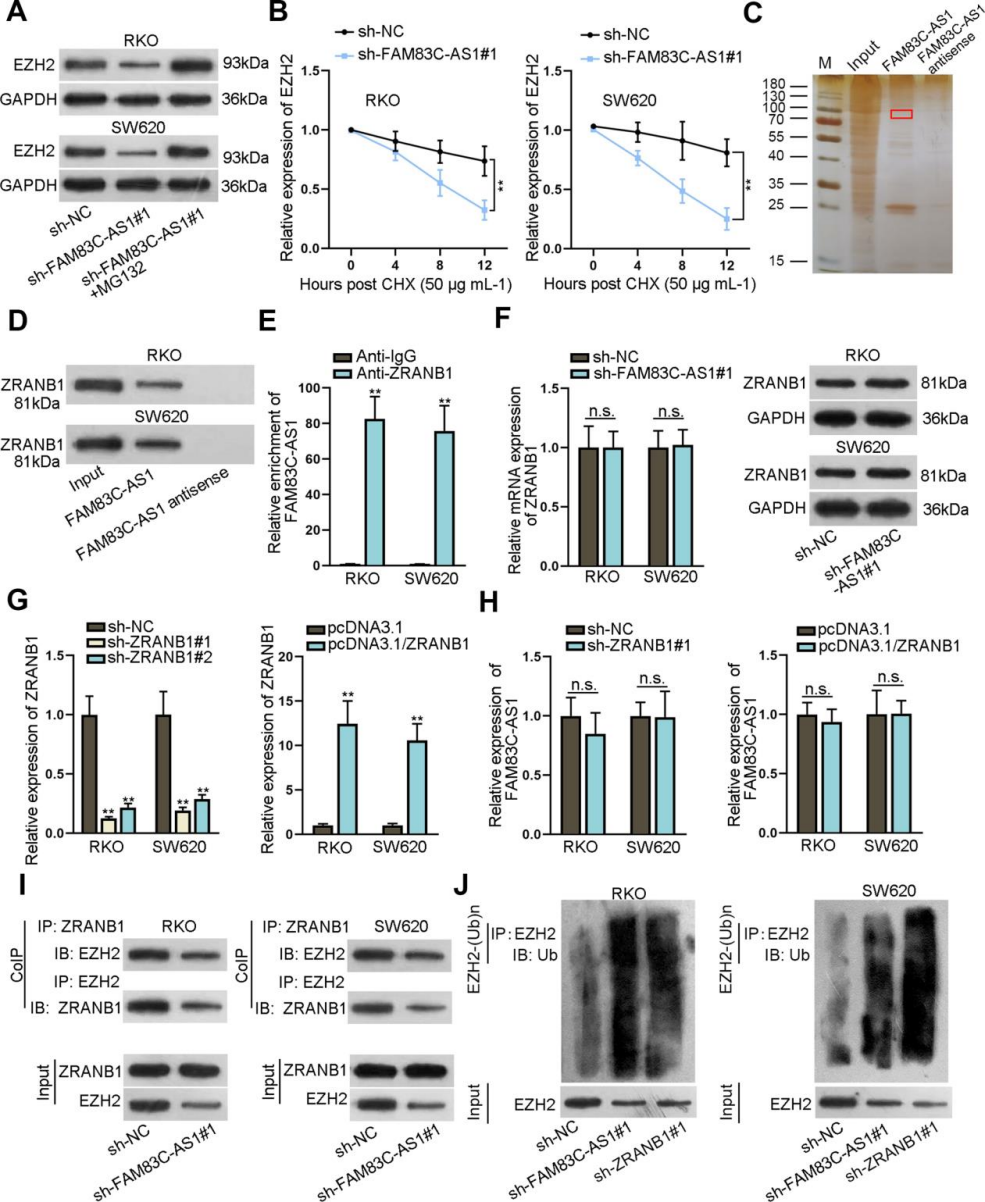
**FAM83C-AS1 stabilized EZH2 protein by binding to ZRANB1 in CRC.** (**A**) EZH2 protein expression was analyzed using western blotting analysis in different groups. (**B**) After treatment with CHX (50 μg mL^-1^), EZH2 protein stability was evaluated in RKO and SW620 cells transfected with sh-FAM83C-AS1#1 or sh-NC. (**C**, **D**) The binding capacity between FAM83C-AS1 and ZRANB1 was measured using RNA pull-down assay. (**E**) RIP assays further proved that FAM83C-AS1 could bind to ZRANB1 in RKO and SW620 cells. (**F**) mRNA and protein expression of ZRANB1 in RKO and SW620 cells transfected with sh-FAM83C-AS1#1 or sh-NC were examined using RT-qPCR and western blotting analysis. (**G**) The efficiency of ZRANB1 knockdown or overexpression was assessed using RT-qPCR analysis. (**H**) FAM83C-AS1 expression in transfected cells was detected by RT-qPCR. (**I**) CoIP assay was adopted for analyzing the effect of FAM83C-AS1 depletion on the binding of ZRANB1 to EZH2. (**J**) The relationship between EZH2 ubiquitination and FAM83C-AS1 or ZRANB1 was analyzed in different groups. ^**^P < 0.01. n.s.: no significance.

To explore the post-transcriptionally mechanism of FAM83C-AS1 in modulation of EZH2 expression, we treated cells with MG132, a kind of proteasome inhibitor. As shown in Figure 4A, EZH2 protein expression was decreased along with the downregulation of FAM83C-AS1 in RKO and SW620 cells. Reciprocally, the inhibitory effects of FAM83C-AS1 on EZH2 expression was attenuated by the addition of MG132 (Figure 4A). We then treated the RKO and SW620 cells with CHX and found that knockdown of FAM83C-AS1 decreased protein stability of EZH2 (Figure 4B). Previous studies have revealed that ZRANB1 binding to EZH2 resulted in EZH2 deubiquitination and thereby stabilized EZH2 protein in breast cancer [25]. Herein, we speculated that FAM83C-AS1 might regulate EZH2 protein stability through binding to ZRANB1. The result of RNA pull-down assay demonstrated the binding capacity between FAM83C-AS1 and ZRANB1 (Figure 4C and 4D). Subsequent RIP assays further validated that FAM83C-AS1 bound to ZRANB1 in RKO and SW620 cells (Figure 4E). We next transfected with sh-FAM83C-AS1#1 into RKO and SW620 cells, and we found that no significant changes in the expression of mRNA and protein expression of ZRANB1 (Figure 4F). Efficiency of ZRANB1 knockdown or overexpression in RKO and SW620 cells was determined using RT-qPCR and the result was satisfactory (Figure 4G). Additionally, enforced expression or knockdown of ZRANB1 had no obvious effefct on the expression of FAM83C-AS1 (Figure 4H). Moreover, CoIP assay indicated that FAM83C-AS1 depletion inhibited the association of ZRANB1 with EZH2 (Figure 4I). More importantly, downregulation of FAM83C-AS1 or ZRANB1 in RKO and SW620 cells enhanced the EZH2 ubiquitination (Figure 4J). Our data thus demonstrate that FAM83C-AS1 binds to ZRANB1 and stabilizes EZH2 protein in CRCs.

### FAM83C-AS1-EZH2-SEMA3F axis exacerbates CRC progression

**Figure 5:**
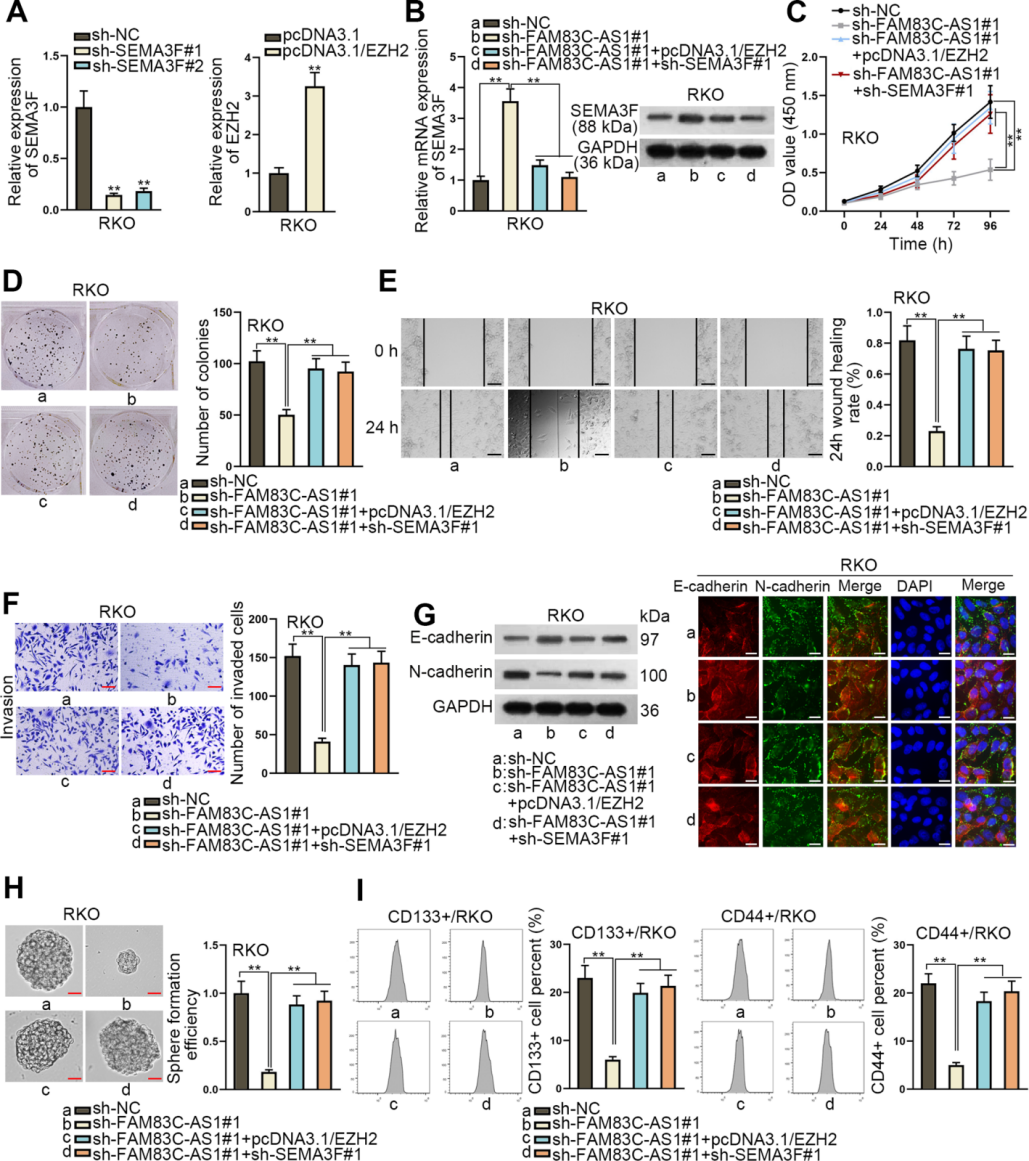
**FAM83C-AS1 mediated CRC progression by stabilizing EZH2 protein to decrease SEMA3F expression.** (**A**) The efficiency of EZH2 overexpression or SEMA3F knockdown was estimated using RT-qPCR. (**B**) SEMA3F expression in transfected cells was examined using RT-qPCR and western blot assay. (**C**, **D**) The proliferation ability of transfected cells was analyzed using CCK-8 and colony formation assays. (**E**, **F**) Wound healing and transwell assays were used to evaluate the migrating and invading abilities of tumor cells in different groups. (**G**) EMT process in transfected cells was measured using western blotting analysis and immunofluorescence assay. (**H**) The sphere formation ability of transfected cells was assessed using sphere formation assay. (**I**) Cell stemness in different groups was analyzed using flow cytometer analysis. ^**^P < 0.01.

To further explore the molecular mechanism of FAM83C-AS1 in CRC progression, we initially knocked down SEMA3F and overexpressed EZH2 in RKO cells (Figure 5A). As illustrated in Figure 5B, EZH2 upregulation or SEMA3F depletion led to the decline of SEMA3F expression. After overexpression of EZH2 or inhibition of SEMA3F, the stimulatory effects of FAM83C-AS1 on the proliferation of RKO cells was blocked (Figure 5C and 5D). Moreover, overexpression of EZH2 or SEMA3F deficiency limited the invasive and migratory capacities of RKO cells in presence of FAM83C-AS1 (Figure 5E and 5F). In addition, western blot and immunofluorescence assay revealed that EZH2 upregulation or SEMA3F depletion countervailed the restraining effect of FAM83C-AS1 deficiency on EMT process (Figure 5G). Besides, upregulated EZH2 or downregulated SEMA3F counteracted the suppressive effect on cell sphere formation ability mediated by FAM83C-AS1 depletion (Figure 5H). Furthermore, flow cytometeric analysis suggested that the inhibitory effect on cell stemness induced by downregulation of FAM83C-AS1 were rescued by EZH2 overexpression or SEMA3F knockdown (Figure 5I). To conclude, our data thus demonstrate that FAM83C-AS1 contributes to CRC progression by stabilizing EZH2 expression and consequently impairing SEMA3F expression.

### FAM83C-AS1 promotes colorectal cancer development by inhibiting SEMA3F expression

**Figure 6:**
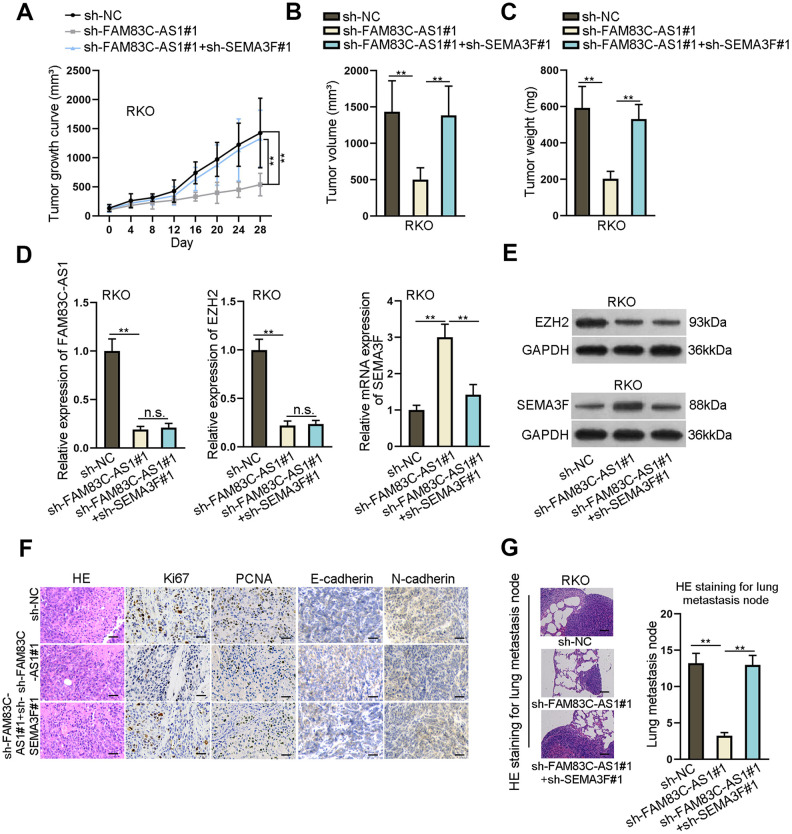
**FAM83C-AS1 promotes tumorigenesis of CRC *in vivo* by inhibiting SEMA3F expression.** (**A**–**C**) Transfected RKO cells were subcutaneously injected into nude mice. Tumor growth curve was obtained by recording tumor volume every 4 days. After 28 days, mice were sacrificed for further evaluation of tumor volume and weight. (**D**) Expression of FAM83C-AS1, EZH2 and SEMA3F in different groups was detected using RT-qPCR. (**E**) Protein expression of EZH2 and SEMA3F in different groups was detected via western blotting analysis. (**F**) Expression of Ki67, PCNA, E-cadherin and N-cadherin in different groups was analyzed using immunohistochemistry assay. (**G**) HE staining for metastasis nodes in lungs was used for evaluating cell metastasis in different groups. ^**^P < 0.01. n.s.: no significance.

To investigate the biological function of FAM83C-AS1 *in vivo*, we subcutaneously injected the FAM83C-AS1-silencing cells or control cells into nude mice. As shown in Figure 6A–6C, downregulation of FAM83C-AS1 restricted tumor growth as relative to control cell did. Moreover, downregulation of SEMA3F rescued the suppressive effect of FAM83C-AS1 on tumor growth, volume and weight (Figure 6A–6C). Besides, rather than upregulation of SEMA3F expression, silencing of FAM83C-AS1 also triggered downregulation of EZH2 in a SEMA3F-indepdent manner (Figure 6D, 6E). Moreover, immunohistochemistry assay revealed that downregulation of SEMA3F countervailed the effect of FAM83C-AS1 sponging on the expression of Ki67, PCNA, E-cadherin and N-cadherin, indicating that SEMA3F depletion promoted tumor growth and EMT process (Figure 6F). To investigate the role of FAM83C-AS1 in cancer metastasis, we intravenously injected mice with sh-FAM83C-AS1 cells, dual-silencing FAM83C-AS1 and SEMA3F cells and control cells, respectively. As shown in Figure 6G, HE staining for metastasis nodes in lungs revealed that, compared with control cells, silencing of FAM83C-AS1 limited the accounts of metastasis nodes, which could be rescued by FAM83C-AS1 knockdown. To sum up, our data demonstrate that FAM83C-AS1 elicits the oncogenic effects on CRC *in vivo* via downregulating the expression of SEMA3F.

## DISCUSSION

CRC remains a lethal malignancy causing numerous cancer related deaths worldwide, and its pathogenesis involves malignant transformation of epithelial cells in the colon or rectum [1]. Enhanced survival capability of tumor cells is considerably important for the biological processes of CRC tumorigenesis and progression [26]. Besides, tumor metastasis, the biological course enabling tumor cells invade into remote organs and tissues, remains a stumbling block in the attempts to cure patients with advanced CRC [7, 8]. Numerous studies have focused on exploring the molecular mechanisms underlying CRC development and progression, particularly the CRC-related genes. Mounting evidence has confirmed that RNA molecules are involved in the tumorigenesis and progression of CRC. For instance, lncRNA PURPL suppresses basal p53 expression and drives CRC tumorigenicity [27]. LncRNA BLACAT1 predicts poor prognosis and promotes cell proliferation through epigenetically silencing p15 in CRC tumor cells [28]. LncRNA SNHG17 is associated with poor prognosis of CRC and promotes CRC cell proliferation via epigenetically silencing P57 [29]. LncRNA CRNDE contributes to proliferation of CRC cells through epigenetically silencing DUSP5/CDKN1A [30]_._ Although SEMA3F has been reported to have inhibitory effect on CRC tumor cell proliferation, metastasis and stemness [23, 24], knowledge of the regulatory mechanism behind SEMA3F expression in CRC remains extremely limited. This study is the first attempt of its kind to explore the regulatory effect of lncRNA on SEMA3F expression in mediating CRC tumorigeness and progression. Based on the results obtained from this study, lncRNA FAM83C antisense RNA 1 (FAM83C-AS1) was found to be highly expressed and negatively correlated with SEMA3F in CRC tumor tissues and cells. Knockdown of FAM83C-AS1 suppresses CRC tumor cell proliferation, migration, invasion and stemness.

Previous studies have revealed that lncRNAs were involved in modulation of gene transcription. For example, lncRNA GHET1 indicates poor prognosis and facilitates cell proliferation through silencing KLF2 expression in hepatocellular carcinoma [31]. LncRNA HOXB13-AS1 exacerbates glioma progression by regulating HOXB13 gene methylation through interacting with EZH2 [22]. LncRNA HOTAIR motivates the self-renewal of leukemia stem cells by epigenetically silencing p15 [32]. More importantly, recent studies have clarified that lncRNA can mediate CRC progression by epigenetically repressing target gene expression [33, 34], which aroused our interest and formed the basis of the following explorations. In the current study, we demonstrate that FAM83C-AS1 transcriptionally regulate SEMA3F expression in CRC tumor cells. FAM83C-AS1 depletion led to decrease in histone H3 lysine 27 (H3K27) trimethylation (H3K27me3) of SEMA3F promoter. Besides, enhancer of zeste 2 polycomb repressive complex 2 subunit (EZH2) could bind to SEMA3F promoter and FAM83C-AS1. Briefly, FAM83C-AS1 promoted H3K27me3 of SEMA3F promoter through binding to EZH2, thereby inhibiting SEMA3F expression.

Based on the result of a previous study, we found that binding of zinc finger RANBP2-type containing 1 (ZRANB1) to EZH2 led to EZH2 deubiquitination, thereby stabilizing EZH2 protein expression in breast cancer [25]. Taken along with the results from this study that FAM83C-AS1 augmented EZH2 protein expression but not mRNA expression in CRC, we proceeded to investigate the underlying mechanism and boldly speculated that FAM83C-AS1 might stabilize EZH2 protein expression through binding to ZRANB1 in CRC tumor cells. Based on molecular mechanism related assays, we concluded that FAM83C-AS1 binding to ZRANB1 promoted EZH2 deubiquitination and thereby inhibited EZH2 downregulation, which led to the enhanced protein stability of EZH2 in CRC tumor cells. After the regulatory effect of FAM83C-AS1 in CRC tumor cells was studied *in vitro*, in vivo assays were successively conducted, which further manifested that FAM83C-AS1 contributed to CRC tumor growth and metastasis by inhibiting SEMA3F expression.

In conclusion, FAM83C-AS1 epigenetically suppressed SEMA3F through stabilizing EZH2 protein and thereby promoting CRC cell proliferation, metastasis and stemness (Supplementary Figure 1). Our findings thus provide evidence of the oncogenic role of FAM83C-AS1s in CRC tumorigenesis and progression both in vitro and in vivo, and shed light on developing promising therapeutic agents for treatment of CRC.

## MATERIALS AND METHODS

### Patients and samples

A total of 70 colorectal cancer tissues as well as their matched adjacent normal tissues were acquired from CRC patients who were received surgical resections during the surgery. All the patients provided written informed consents and the study protocol was approved by the ethics committee of the Harbin Medical University Cancer Hospital. No participant was treated with radiotherapy or chemotherapy before the surgery. All the paired CRC samples were snap-frozen at -80^o^C in liquid nitrogen for subsequent analysis.

### Microarray analysis

Total RNA were extracted from the 3 pairs of CRC tumor tissue and adjacent normal tissue specimens according to the instruction of TRIzol Reagent (Invitrogen, Carlsbad, CA) for sample preparation, which was followed by microarray hybridization. The lncRNAs with differential expression were screened out as per the criteria of log2 fold change > 2 and adjusted *p* < 0.01, and the results were presented using the Heat map.

### Cell lines and reagents

Human CRC cell lines (SW480, HT29, SW620, RKO) and normal human colonic epithelial cell lines (HCoEpiC) were all procured from ATCC (Manassas, VA); and the cells were separately incubated at 37^o^C with 5% CO_2_. DMEM medium (Gibco, Grand Island, NY) supplemented with 10% FBS (Gibco) in the presence of 1% antibiotics. And the RKO and SW620 cells were treated with 50 mg/mL cycloheximide (CHX) and 10 mM of MG132 (all from ENZO Life Sciences, Farmingdale, NY), respectively.

### Total RNA extraction and RT-qPCR

Total RNAs were extracted from the specimens using the Trizol method (Invitrogen) according to the instruction for use provided by the supplier and cDNA was synthesized using the reverse transcription kit (Applied Biosystems, Carlsbad, CA). RT-qPCR detection was performed using the Power SYBR^®^ Green Master mix purchased from Applied Biosystems. The mRNA expression levels of the tested genes relative to GAPDH were determined using the 2^-ΔΔCt^ method.

### Subcellular fractionation

Subcellular fractionation assay was conducted to obtain cell nucleus and cell cytoplasm using the cytoplasmic and nuclear RNA purification kit (Norgen, Thorold, ON, Canada). Expression levels of FAM83C-AS1, GAPDH and U6 in cytoplasmic and nuclear fractions of CRC cells were determined using the RT-qPCR method.

### Fluorescence in situ hybridization assay (FISH)

Hybridization with air-dried CRC cell samples was performed using the specific probes for FAM83C-AS1-FISH assay (from Ribobio Guangzhou, China) as per the protocol. Samples were counterstained with DAPI and viewed under a microscope (Leica, Wetzlar, Germany).

### Transfection

The specific short hairpin RNAs (shRNAs) and small hairpin RNAs-negative control (shRNAs-NC), purchased from Genepharma Company (Shanghai, China), were used to silence the expression of FAM83C-AS1, EZH2 and SEMA3F in RKO and SW620 cells following transfection with Lipofectamine2000 (Invitrogen) for 48 h. In addition, the pcDNA3.1 vectors and control vectors (NC, Genepharma) were employed to overexpress EZH2 and ZRANB1.

### Western blot assay

After total protein were extracted using radio immunoprecipitation assay (RIPA) lysis buffer, protein samples were subjected to 10% sodium dodecyl sulfate-polyacrylamide gel electrophoresis (SDS-PAGE) gel electrophoresis and transferred onto polyvinyl difluoride (PVDF) membranes. After blocked with 5% non-fat milk, the membranes were incubated with the primary antibodies and HRP-conjugated secondary antibodies, purchased from Abcam (Cambridge, MA) after dilution. GAPDH was used as an internal standard. After samples were washed with TBST for several times, protein bands were visualized using an enhanced chemiluminescence detection reagent (Thermo Fisher Scientific, Rochester, NY).

### CCK-8 assay

Samples of CRC cells were cultured at logarithmic growth phase and inoculated to 96-well plates. After the samples were added with 10 μl of Cell Counting Kit-8 (CCK-8) detection solution and incubated for 2 h, absorption was monitored at 450 nm by a microplate reader.

### Colony formation assay

CRC cells were prepared and inoculated to the 6-well plates and were incubated for 14 days. Then, the cells were fixed in 4% paraformaldehyde and stained with 0.5% crystal violet, and finally the colony numbers were counted.

### Wound-healing assay

RKO and SW620 cells seed in 6-well plates were transfected and cultured until the cells grew to confluent. Then the cells were wounded by scratching with 200 μL sterile plastic pipette tips vertically against the well, followed by rinsing in PBS buffer. The wounds were examined at time 0 and 24 h to assess cell migration.

### Transwell assay

Tumor cell invasion and migration were analyzed using a Matrigel-coated transwell chamber (Corning Co, Corning, NY) as per the instruction. And 5000 CRC cell samples were added to the upper chamber with serum-free medium, with conditioned medium placed to the lower chamber. Invading cells were fixed and stained with crystal violet solution for 24 h. Five random fields were selected for cell counting.

### Immunofluorescence (IF) test

After culturing for 24 h, CRC cells were transferred onto coverslips and fixed for 30 min; and were incubated first with specific antibodies against E-cadherin and N-cadherin overnight and then with FITC conjugated secondary antibody (Abcam). After rinsing for several times, fluorescent staining was used to observe the fluorescence intensity under a fluorescence microscope (Olympus, Tokyo, Japan). DAPI-stained nuclei were analyzed for DNA content.

### Sphere formation assay

CRC cells were seeded in 96-well ultralow attachment plates (Corning) with sphere formation medium at a density of 10 cells per well and incubated for 7 days. The number of sphere cells formed was then counted.

### Flow cytometer analysis

Cell samples cultured in 1% BSA were incubated with 1 μl PE-conjugated anti-human CD133 or CD44 antibody at 4°C for 1 h. After washing, the cells were analyzed with flow cytometer using the FACSCalibur system (BD Biosciences, San Jose, CA).

### Luciferase reporter assay

CRC cells were inoculated to 24-well plates and prepared for co-transfection with pGL3-vetcor (Promega Corporation, Fitchburg, WI) covering SEMA3 promoter, pRL-TK-Renilla (Promega) and silencing the expression of plasmids of FAM83C-AS1 or EZH2. And after 48 h, luciferase reporter assay of SEMA3 promoter was performed using the dual-luciferase reporter assay system (Promega).

### Chromatin immunoprecipitation (ChIP) assay

The cross-linked chromatin was sheared into 200-1000 fragments to allow for immunoprecipitation with specific antibodies. Normal mouse IgG was used as the negative control. And finally, expression of mRNA was measured using RT-qPCR.

### RNA-inding protein immunoprecipitation (RIP) assay

1×10^7^ RKO and SW620 cells were harvested with lysis buffer and immunoprecipitation was carried out with antibodies using anti-IgG as negative control. After mixing with beads, the precipitates were determined using RT-qPCR.

### Chromatin immunoprecipitation (assay)

After lysing in the immunoprecipitation (IP) lysis buffer, cell lysates were reaped and cultured with antibodies and negative control of anti-IgG at 4^o^C, followed by incubation with beads for 2 h. The eluted protein samples were determined using western blot analysis.

### RNA pull-down assay

The protein lysates derived from CRC cells were harvested and mixed with biotinylated RNA (or RNA antisense) and beads for 2 h. The RNA-protein mixed solution was analyzed using sliver staining or western blot method.

### Animal study

The study was approved by the Institutional Animal Care and Use Committee of the Harbin Medical University Cancer Hospital. Male nude mice aged 6-8 weeks (National Laboratory Animal Center, Beijing, China) were used for the study. Transfected CRC cells were transplanted into the mice via subcutaneous injection for 28 days to the xenograft model and observe *in vivo* tumor growth in the nude mice. Tumor volumes were measured every 4 days in all mice. Then the mice were sacrificed at different time points after the transplantation by injection ofvia tail vein. And the lungs were excised to evaluate the metastatic nodules on the surface of the lungs using hematoxylin-eosin (HE) staining. All the tumor tissues were snap frozen in liquid nitrogen and then stored in a refrigerator at -80°C until analysis.

### Immunohistochemistry (IHC) staining

The frozen tumor tissues were thawed and fixed in 4 % paraformaldehyde and embedded in paraffin. After cutting into small pieces, the consecutive sections measured 4 μm in thickness were used for IHC staining with antibodies against Ki67, PCNA, E-cadherin and N-cadherin (Abcam).

### Statistical analysis

All data was presented as mean ± SD determined by three independent experiments and processed using the SPSS.19.0 software (IBM Corp., Armonk, NY) or PRISM 6 (GraphPad, San Diego, CA). Intergroup difference was analyzed using student’s *t* test and one-way ANOVA, and *p* < 0.05 was considered as statistically significant. Correlation analysis was examined with Pearson’s χ2 analysis.

## Supplementary Material

Supplementary Figure 1

Supplementary Table 1
