# Supplementary material for: The oncogenic role of LncRNA FAM83C-AS1 in colorectal cancer development by epigenetically inhibits SEMA3F via stabilizing EZH2
Source: Aging (Albany NY). 2020 Oct 27;12(20):20396–412. doi: 10.18632/aging.103835 (PMC7655168; doi:10.18632/aging.103835)
Supplement: Supplementary Figure 1 [file aging-12-103835-s001..pdf]

SUPPLEMENTARY FIGURE

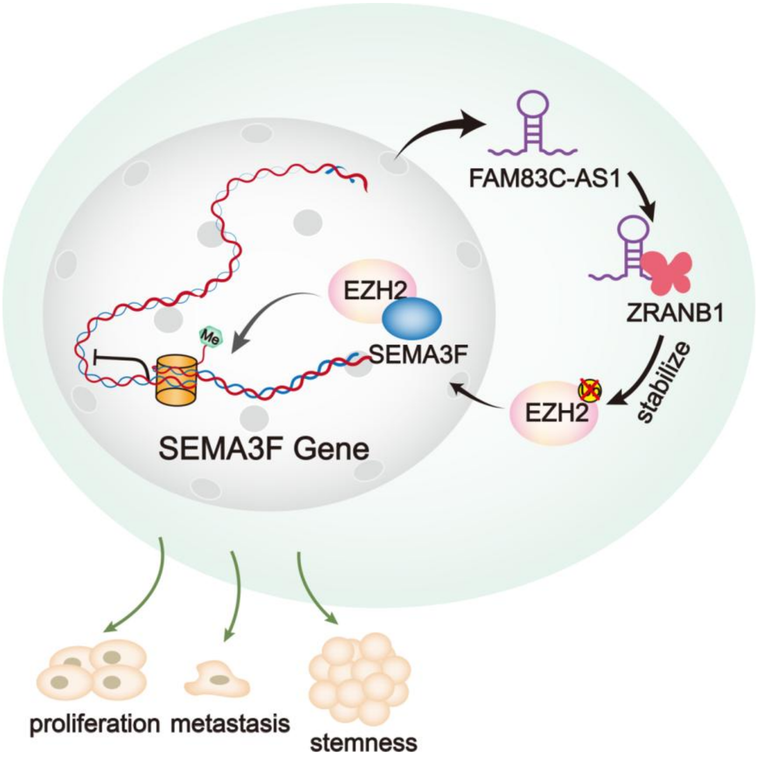

Supplementary Figure 1. A graphical abstract of the regulatory mechanism of FAM83C-AS1 in CRC was shown in Figure 1.
